# Speech Recognition with an fMRISNN Constrained by Human Functional Brain Networks: A Study of Enhanced MFCC-Driven Sparse Spike Encoding

**DOI:** 10.3390/biomimetics11050302

**Published:** 2026-04-26

**Authors:** Lei Guo, Nancheng Ma, Zhuoxuan Wang, Rumeng Liu

**Affiliations:** 1 Tianjin Key Laboratory of Bioelectromagnetic Technology and Intelligent Health, School of Health Sciences and Biomedical Engineering, Hebei University of Technology, Tianjin 300131, China; 202322902019@stu.hebut.edu.cn (N.M.); 202432903015@stu.hebut.edu.cn (Z.W.); 202532903015@stu.hebut.edu.cn (R.L.); 2 State Key Laboratory of Intelligent Power Distribution Equipment and System, Hebei University of Technology, Tianjin 300401, China

**Keywords:** SNN, fMRI, functional brain network, sparse spike encoding, speech recognition

## Abstract

Spiking neural networks (SNNs) offer inherent advantages in processing temporal information. However, their network topologies are predominantly algorithm-generated, lacking constraints from biological brain connectivity, which limits their bio-plausibility. In our previous work, we constructed a spiking neural network (SNN) by incorporating the topological structure of functional brain networks derived from fMRI data of healthy subjects and proposed an fMRISNN model. This model was further employed as the reservoir layer of a liquid state machine (LSM) to build a speech recognition framework. In this framework, the Lyon ear model and the BSA were used to encode speech signals into spike sequences; however, this approach suffers from high computational cost and limited adaptability to temporal variations. To address these limitations, we propose an enhanced Mel-frequency cepstral coefficient (MFCC)-driven sparse spike encoding method. For the speech recognition task, we systematically compare the two preprocessing pipelines in terms of spike number, spike sparsity, encoding time, and downstream speech recognition performance. Experimental results show that the proposed method generates substantially fewer spikes, achieves markedly higher sparsity, and requires significantly less encoding time, while maintaining nearly the same recognition accuracy under the same LSM-based framework. These findings indicate that improved speech input representation can enhance the computational efficiency of SNN-based speech recognition without compromising recognition capability. In addition, the fMRISNN model significantly outperforms several baseline models with algorithmically generated topologies. Compared with mainstream models reported in the literature, although the deep convolutional neural network (CNN) still achieves higher absolute recognition accuracy, the fMRISNN exhibits clear advantages in terms of model parameter size and theoretical energy efficiency.

## 1. Introduction

The biological brain provides important inspiration for the development of artificial intelligence. As one of the most representative models in brain-inspired computing, the spiking neural network (SNN) exhibits natural advantages in processing temporal information because computation is carried out through dynamic interactions among spikes, neurons, and synapses [1,2]. Compared with conventional artificial neural networks, SNNs encode and transmit information in an event-driven manner, which makes them especially suitable for time-varying signals such as speech [1,3]. Nevertheless, although many existing SNNs attempt to mimic neuronal dynamics, their network topologies are still mainly generated by algorithmic rules [4], such as regular, random, small-world, or scale-free structures [5,6,7]. This means that the structural organization of these models often remains insufficiently constrained by biological brain connectivity. Recent advances in neuroimaging provide a new opportunity to address this limitation. Functional magnetic resonance imaging (fMRI) makes it possible to estimate large-scale functional brain networks (FBNs) in the human brain [8,9]. In fMRI, the measured signal is the blood oxygenation level-dependent (BOLD) response, which reflects hemodynamic changes associated with neural activity [10,11]. From the perspective of brain-inspired modeling, FBNs derived from fMRI offer biologically grounded macro-scale structural priors that may guide the construction of artificial neural systems. Such priors are particularly attractive because they capture stable communication patterns among brain regions and can potentially serve as an architectural scaffold for neural computation. This perspective motivates the integration of brain-network topology into the design of SNNs.

Among SNN models for temporal processing, the liquid state machine (LSM) is especially suitable for speech recognition [12]. The reservoir in an LSM projects temporal inputs into a rich high-dimensional dynamic space, and the quality of this transformation depends strongly on the internal network topology. Previous studies have shown that the topology of the reservoir significantly influences classification performance, generalization ability, and information transmission efficiency in temporal tasks [13,14,15]. These observations suggest that constraining the topology of an SNN with FBNs may provide a promising route toward both higher bio-plausibility and improved performance. On this basis, our previous study constructed an fMRISNN constrained by the topology of human FBNs, demonstrating that an LSM based on fMRISNN can achieve favorable speech recognition performance while exhibiting advantages in robustness and information transmission [16,17,18]. In this speech recognition framework, speech preprocessing plays a critical role because it determines the temporal statistics, sparsity, and discriminability of the spike sequences delivered to the reservoir, thereby directly affecting the performance of recognition based on the SNN. In conventional speech processing, the Lyon ear model is often adopted as a biologically inspired auditory front-end. By cascading asymmetric resonators, half-wave rectification, and automatic gain control, it simulates cochlear frequency selectivity, compression, and adaptation, thereby preserving important spectro-temporal information in speech signals [19,20]. On this basis, the Bens Spiker algorithm (BSA) is commonly used to convert continuous auditory representations into spike trains [21]. Accordingly, our previous framework employed the Lyon ear model and the BSA to encode speech signals into spike sequences [16]. Although this biologically inspired pipeline has been widely used, it still presents several limitations. Its encoding stage depends on the joint design of finite impulse response (FIR) filters and threshold parameters, making parameter selection relatively complex. Moreover, previous studies have shown that BSA is less effective in representing abrupt temporal changes, such as step-like transitions [22,23]. These limitations suggest that the conventional Lyon ear model–BSA pipeline still leaves room for improvement in terms of computational efficiency, spike sparsity, and adaptability to temporal variation.

To address this issue, this paper proposes an enhanced Mel-frequency cepstral coefficient (MFCC)-driven sparse spike encoding method for speech recognition. Specifically, the proposed method integrates enhanced MFCC-based dynamic feature extraction with sparse spike conversion, temporal alignment, and normalization. In addition, the present study explicitly compares the proposed preprocessing pipeline with the conventional Lyon ear model + BSA pipeline under the same fMRISNN-based speech recognition framework. This comparison is carried out both at the encoding level and at the downstream recognition level. The main contributions of this study can be summarized as follows:

1. An enhanced MFCC-driven sparse spike encoding method is introduced as a preprocessing extension to the existing fMRISNN-based speech recognition framework.
2. A controlled comparison is conducted between the proposed preprocessing pipeline and the conventional Lyon ear model + BSA pipeline at both the encoding level and the downstream recognition level under the same fMRISNN architecture.
3. Experimental results show that the proposed method reduces spike number, increases spike sparsity, and shortens encoding time while maintaining nearly the same speech recognition accuracy.
4. The fMRISNN reservoir remains competitive with baseline models using algorithmically generated topologies. Compared with the deep convolutional neural network (CNN), although it still achieves higher absolute recognition accuracy, the fMRISNN exhibits clear advantages in terms of model parameter size and theoretical energy efficiency.

The rest of this paper is organized as follows. Section 2 describes the construction of the fMRISNN. Section 3 presents the enhanced MFCC-driven sparse spike encoding method. Section 4 introduces the fMRISNN-based speech recognition framework and presents the experimental results. Section 5 discusses the speech recognition mechanism of the fMRISNN. Finally, Section 6 concludes the paper.

## 2. Construction of fMRISNN

The fMRISNN used in this study follows our previously established framework [16]. To ensure a controlled investigation, we preserve the major structural components of that framework, including the FBN-constrained topology, the leaky integrate-and-fire (LIF) neuron model, and the synaptic plasticity mechanism with transmission delay. This design choice allows us to isolate the effect of the revised speech preprocessing and spike encoding pipeline and to examine whether improvements in input representation can enhance speech recognition under the same biologically constrained reservoir structure.

### 2.1. The Topological Structure of fMRISNN

The network topology of the fMRISNN constructed in this study is based on an FBN acquired from fMRI data of the human brain. The fMRI data used was extracted from the Neuroimaging Informatics Tools and Resources Collaboratory (NITRC) database [24], encompassing 10 healthy subjects (5 females, 5 males) aged 20–25 years (mean 21.60, standard deviation 1.83). Following graph theory [25], we construct an FBN by mapping brain regions to nodes, their functional connections to edges, and the connection strengths to edge weights. Using subject 4546 as an example, the network generation process is illustrated below.

#### 2.1.1. Network Nodes

The fMRI data were parcellated into 90 brain regions using the Anatomical Automatic Labeling (AAL) standard brain atlas [26], with each region defined as a network node. Before network construction, the fMRI data were preprocessed to ensure the reliability of the extracted regional BOLD time series. The fMRI data were acquired with a repetition time (TR) of 1.5 s and an echo time (TE) of 28 ms. Slice timing correction was first performed for 40 slices, using an acquisition interval of 1.4625 s and the 21st slice as the reference. Head motion correction was then applied by realigning all volumes to the mean image. Subsequently, spatial normalization was performed using TPM.nii, and all images were resampled to 2 mm × 2 mm × 2 mm. Spatial smoothing was carried out using a 6 mm × 6 mm × 6 mm Gaussian kernel. Finally, temporal band-pass filtering (0.01–0.1 Hz; sampling frequency = 0.67 Hz) was applied to reduce non-neuronal noise. The preprocessed regional mean BOLD time series extracted from the 90 brain regions defined by the AAL atlas were then used to construct the functional brain networks.

#### 2.1.2. Network Edges

**Figure 1:**
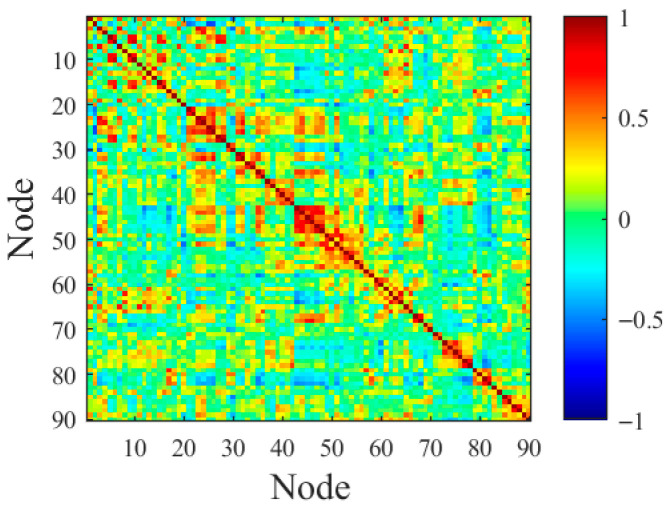
Correlation coefficient matrix of the neuron node.

Functional connectivity between node pairs was quantified using the Pearson correlation coefficient calculated from the corresponding BOLD time series. The formula for calculating the Pearson correlation coefficient R is as follows:

(1) $$R_{ij}\;=\;\frac{\sum_{t=1}^{T}\left(x_{i}\left(t\right)-{\bar{x}}_{i}\right)\left(x_{j}\left(t\right)-{\bar{x}}_{j}\right)}{\sqrt{\sum_{t=1}^{T}{\left(x_{i}\left(t\right)-{\bar{x}}_{i}\right)}^{2}\sum_{t=1}^{T}{\left(x_{j}\left(t\right)-{\bar{x}}_{j}\right)}^{2}}}$$

where $R_{ij}$ represents the Pearson correlation coefficient between node i and node j. When the absolute value of the correlation coefficient approaches 1, it indicates a strong functional connection between the corresponding brain region nodes. The means of the BOLD signal time series for node i and node j at time t are denoted as $x_{i}\left(t\right)$ and $x_{j}\left(t\right)$, respectively, and their means over the entire scanning duration T are denoted as $x_{i}\left(t\right)$ and $x_{j}\left(t\right)$, respectively. By calculating the Pearson correlation coefficients between neural nodes, a correlation coefficient matrix is found, as shown in Figure 1.

**Figure 2:**
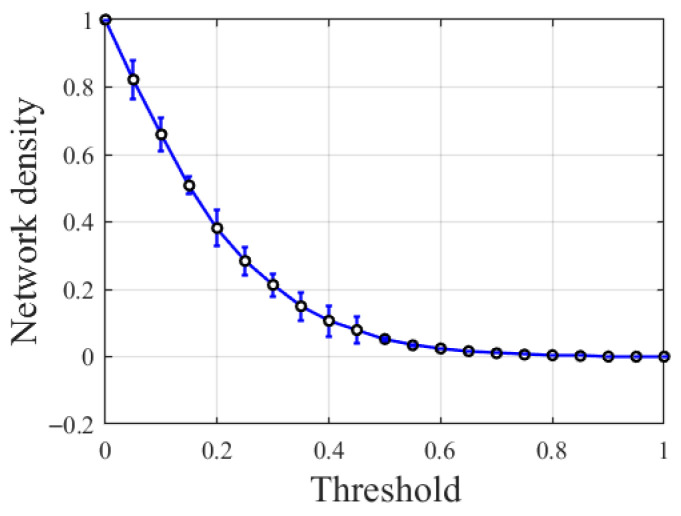
Network density corresponding to different $X_{th}$ values.

To construct the topology of our proposed model, an appropriate threshold $X_{th}$ is required to define whether an edge exists between two nodes. When the functional connection strength is greater than $X_{th}$, a connection exists between the nodes; otherwise, there is no connection. Research data indicate that the density range of biological brain networks typically falls between 3.6% and 39.3% [27]. To ensure that the model structure aligns with the density characteristics of biological brain networks, this study determines the connection relationships between network nodes by setting an appropriate threshold $X_{th}$. Here, network density is defined as the ratio of the actual number of connection edges to the theoretical maximum possible number of edges. Using a step size of 0.05, the network densities corresponding to different $X_{th}$ thresholds within the [0, 1] interval were calculated. Figure 2 shows the mean and standard deviation of network densities for 10 subjects under different $X_{th}$ values.

As shown in Figure 2, when 0.2 ${\leq X}_{th}\leq$ 0.5, the network density ranges between 3.6% and 39.3%, all falling within the density range of biological brain networks. Among these, at $X_{th}$ = 0.3, the corresponding standard deviation is the smallest, indicating the least variation in network density across the 10 subjects. Furthermore, the FBN constructed with $X_{th}$ = 0.3 can more accurately reflect the network characteristics of all subjects.

**Figure 3:**
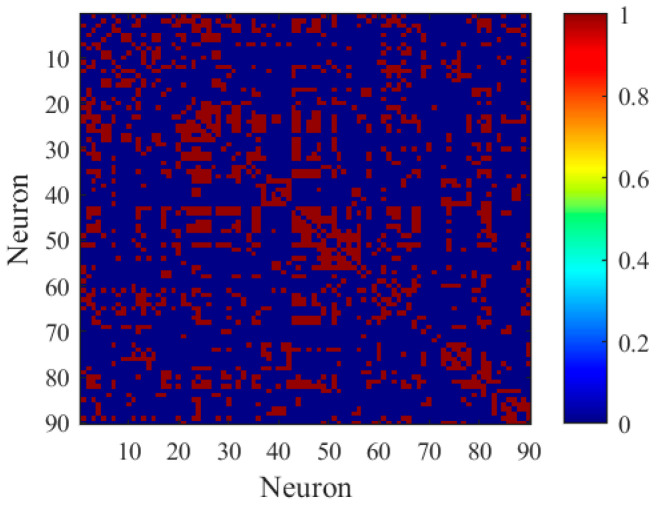
Edge connection matrix (red nodes indicate connections between two neurons; blue nodes indicate no connection).

Therefore, this study determines the appropriate value for $X_{th}$ to be 0.3. Based on this threshold, the edge connection relationships for the FBN were established. Figure 3 shows a schematic diagram of the edge connection matrix for subject 4546.

#### 2.1.3. Network Topology

**Figure 4:**
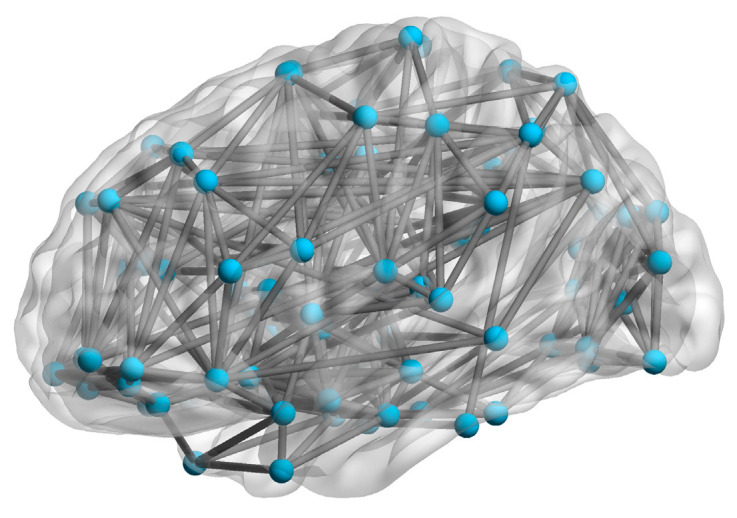
Visualization of FBN topology. (The blue circles represent network nodes, and the gray lines represent connections between nodes).

The topological structure of the FBN was obtained by mapping the distribution and connectivity of its nodes. Figure 4 shows a visualization of the topological structure for subject 4546.

Following the methods described in references [28,29], the small-world (*δ*) and scale-free (*γ*) characteristics of these FBNs were calculated. A network is considered to possess *δ* characteristic when its *δ* value is greater than 1. The average *δ* value for these FBNs was 1.76, confirming their *δ* characteristic. If the degree distribution of a network follows a power-law distribution with an exponent *γ* between 2 and 3, the network exhibits the *γ* characteristic. The average *γ* value for these FBNs was 2.08, indicating that they also possess the *γ* characteristic. Therefore, we adopted a topological structure that exhibits both *δ* and *γ* characteristics as the topology for the proposed model.

### 2.2. The Neuron Model of fMRISNN

This study employs the LIF neuron model as the fundamental computational unit of the fMRISNN. The model uses a concise first-order differential equation to describe the membrane potential dynamics of a neuron, achieving a good balance between computational efficiency and bio-plausibility. The core dynamics of the LIF neuron are described by the following equation:

(2) $$\tau_{m}\frac{dV}{dt}\;=\;-\left(V\left(t\right)-V_{\mathrm{rest}}\right)+R_{m}I_{syn}\left(t\right)$$

where $V\left(t\right)$ represents the membrane potential at time t; $\tau_{m}=R_{m}C_{m}$ is the membrane time constant, which determines the rate of potential decay; $V_{\mathrm{rest}}$ is the resting potential; $R_{m}$ is the membrane resistance; and $I_{syn}\left(t\right)$ is the total input synaptic current.

When the membrane potential $V\left(t\right)$ reaches or exceeds the firing threshold $V_{\mathrm{th}}$, the neuron generates a spike (or action potential), after which the membrane potential is immediately reset:

(3) $$V\left(t\right)\;\geq \;V_{th}\Rightarrow \left\{\begin{array}{c} S\left(t^{\left(f\right)}\right)\;=\;1\\ V\left(t^{\left(f\right)+}\right)\;=\;V_{reset} \end{array}\right.$$

where $t^{\left(f\right)}$ represents the precise firing time of the f-th spike; $t^{\left(f\right)+}$ represents the moment immediately after the spike, at which the membrane potential is reset; $V_{reset}$ is the reset potential, typically satisfying $V_{reset}\leq V_{rest}<V_{th}$. After firing a spike, the neuron enters an absolute refractory period with a duration of $\tau_{\mathrm{ref}}$, during which the membrane potential is clamped at $V_{reset}$ and does not respond to any input. Here, $S\left(t^{\left(f\right)}\right)$ is the spike indicator: $\left(t^{\left(f\right)}\right)$ = 1 marks the occurrence of a spike at the firing time $t^{\left(f\right)}$.

The parameters of the LIF neurons used in this study are listed in Table 1.

### 2.3. The Synaptic Plasticity Model of fMRISNN

We define a synaptic plasticity model with time-delay characteristics [30] as follows:

(4) $$I_{syn}\left(t\right)\;=\;g\left(t\right)r_{g}\left(t\right)\left(E_{syn}-V_{post}\left(t\right)\right)$$

where $I_{syn}$ represents the synaptic current, $E_{syn}$ denotes the synaptic reversal potential, $V_{post}$ is the postsynaptic membrane potential, and g is the synaptic weight. $r_{g}$ indicates the proportion of bound receptors affected by the neurotransmitter concentration *H*, and their relationship is given as follows:

(5) $$\left\{\begin{array}{c} \frac{dr_{g}}{dt}\;=\;\alpha H\left(1-r_{g}\right)-\beta r_{g}\\ H\;=\;{\left[1+\exp\left(-V_{pre}\left(t-\tau \right)\right)\right]}^{-1} \end{array}\right.$$

where α represents the forward rate constant of the neurotransmitter, β denotes the reverse rate constant of the neurotransmitter, $V_{pre}$ is the presynaptic membrane potential, and τ represents the time constant of short-term depression.

The excitatory ($g_{ex})$ and inhibitory $\left(g_{in}\right)$ synaptic weight are updated according to the following rule [31]: When a presynaptic neuron i fails to transmit an action potential to postsynaptic neuron j, the values of $g_{ex}$ and $g_{in}$ exhibit exponential decay, calculated as follows:

(6) $$\tau_{ex}dg_{ex}/dt\;=\;-g_{ex};{\;\tau }_{in}dg_{in}/dt\;=\;-g_{in}$$

where${\;\tau }_{ex}$ and $\tau_{in}$ are the decay constants for $g_{ex}$ and $g_{in}$, respectively. When neuron j receives an action potential from neuron i, the changes in the values of $g_{ex}$ and $g_{in}$ can be described as follows:

(7) $$\left\{\begin{array}{c} g_{ex}\left(t\right)\;=\;g_{ex}\left(t\right)+{\bar{g}}_{ex}\\ {\bar{g}}_{ex}\;=\;w\left(∆t\right)\times g_{max} \end{array};\;w\left(∆t\right)\;=\;\left\{\begin{array}{c} A_{+}\exp\left(∆t/\tau_{+}\right),∆t<0\\ {-A}_{-}\exp\left(∆t/\tau_{-}\right),∆t\geq 0 \end{array}\right.\right.$$

(8) $$\left\{\begin{array}{c} g_{in}\left(t\right)=g_{in}\left(t\right)+{\bar{g}}_{in}\\ {\bar{g}}_{in}=m\left(∆t\right)\times g_{max} \end{array}\right.;\;m\left(∆t\right)=\left\{\begin{array}{c} {-B}_{+}\exp\left(∆t/\tau_{+}\right),∆t<0\\ B_{-}\exp\left(∆t/\tau_{-}\right),∆t\geq 0 \end{array}\right.$$

where ${\bar{g}}_{ex}$ and ${\bar{g}}_{in}$ represent the increments of $g_{ex}$ and $g_{in}$, respectively. $g_{max}$ is the maximum synaptic weight: if ${g>g}_{max}$, it is set to $g\;=g_{max}$; if g<0, it is set to g =0. $w\left(∆t\right)$ and $m\left(∆t\right)$ are modulation functions designed based on the spike-timing-dependent plasticity rule; ∆t denotes the time interval between the presynaptic and postsynaptic spikes. $A_{+}$ and $B_{+}$ are the maximum modulation values for the increase in $g_{ex}$ and $g_{in}$, respectively; $A_{-}$ and $B_{-}$ are the minimum modulation values for the decrease in $g_{ex}$ and $g_{in}$, respectively; and $\tau_{+}$ and $\tau_{-}$ correspond to the neural firing intervals for the increase and decrease in g, respectively.

The synaptic delay in neural systems typically undergoes dynamic regulation within a range of 0.1–40 ms [32]. Therefore, a delay mechanism was incorporated into the synaptic model. The delay values were set according to a random distribution within the [0.1, 40] millisecond range and followed a Poisson distribution. As described in references [30,31], Table 2 lists the parameters and their values for the synaptic plasticity model.

## 3. Enhanced MFCC-Driven Sparse Spike Encoding

**Figure 5:**
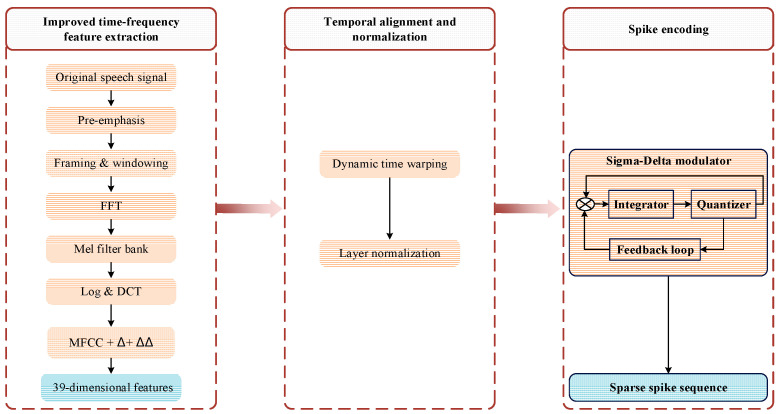
Enhanced MFCC-driven sparse spiking coding method.

Raw speech data, as an analog signal, must be converted into spike trains to be effectively processed by neuron models. In traditional methods, the Lyon ear model is widely employed due to its bio-plausibility and capability to capture spectral features [33]. Combined with the BSA, it enables efficient spike encoding [21]. However, this approach has limitations, including relatively high computational cost and insufficient adaptability to temporal variations, especially in complex acoustic environments [22]. To address these limitations, this study proposes an enhanced MFCC-driven sparse spike encoding method. This method incorporates auditory-inspired mechanisms including the Mel-frequency scale [34], non-uniform filter bank analysis [35], and logarithmic compression [36] during the feature extraction process. Compared with the conventional Lyon ear model + BSA pipeline, the proposed method is designed to improve speech input representation in terms of spike number, sparsity, encoding time, and recognition accuracy within the same fMRISNN-based speech recognition framework. The speech preprocessing pipeline based on the enhanced MFCC-driven sparse spike encoding method is illustrated in Figure 5.

### 3.1. Improved Time–Frequency Feature Extraction

An enhanced MFCC feature extraction scheme is employed, which augments the standard MFCC by incorporating first-order (Δ) and second-order (ΔΔ) dynamic features estimated using a central difference derivative estimator. This combined feature set captures both the static spectral characteristics and the dynamic variation patterns of the speech signal, resulting in a 39-dimensional feature vector (13-dimensional static MFCC + 13-dimensional Δ + 13-dimensional ΔΔ). The feature extraction process can be formally described as follows:

a. Pre-emphasis, framing, and windowing are applied to the speech signal.

b. The Fast Fourier Transform (FFT) magnitude spectrum is computed for each frame.

c. Filtering is performed using a Mel filter bank to obtain the Mel spectrum.

d. The logarithm is taken of the Mel spectrum, followed by Discrete Cosine Transform (DCT).

e. Compute the first-order and second-order dynamic features using a central difference derivative estimator:

(9) $$\Delta c_{t}\;=\;\frac{\sum_{n=1}^{N}n\left(c_{t+n}-c_{t-n}\right)}{2\sum_{n=1}^{N}n^{2}}$$

(10) $$∆∆c_{t}=\frac{\sum_{n=1}^{N}n\left({\Delta c}_{t+n}-{\Delta c}_{t-n}\right)}{2\sum_{n=1}^{N}n^{2}}$$

where $c_{t}$ represents the MFCCs of the t-th frame, ${∆c}_{t}$ represents the first-order dynamic feature, ${∆∆c}_{t}$ represents the second-order dynamic feature, and N is the context window size, typically set to 2.

f. Concatenate the feature vectors:

(11) $$F_{\mathrm{enhanced}}\;=\;{\left[C,\Delta C,\Delta \Delta C\right]}^{T}$$

### 3.2. Temporal Alignment and Normalization

The dynamic time warping (DTW) algorithm is introduced to handle speech signals of variable lengths. It achieves alignment between sequences of different lengths by finding a minimum-cost path. The alignment distance is defined as

(12) $$D\left(i,j\right)=d\left(i,j\right)+min\left\{\begin{array}{c} D\left(i-1,j\right)\\ D\left(i,j-1\right)\\ D\left(i-1,j-1\right) \end{array}\right.$$

where d(i,j) is the feature distance between frame i and frame j.

The aligned feature sequences are then normalized using the layer normalization method:

(13) $$x_{\mathrm{nr}}\;=\;\frac{x-\mu }{\sigma +\epsilon }$$

where μ and σ are the mean and standard deviation of the features, respectively, and ϵ is a small constant to prevent division by zero.

**Figure 6:**
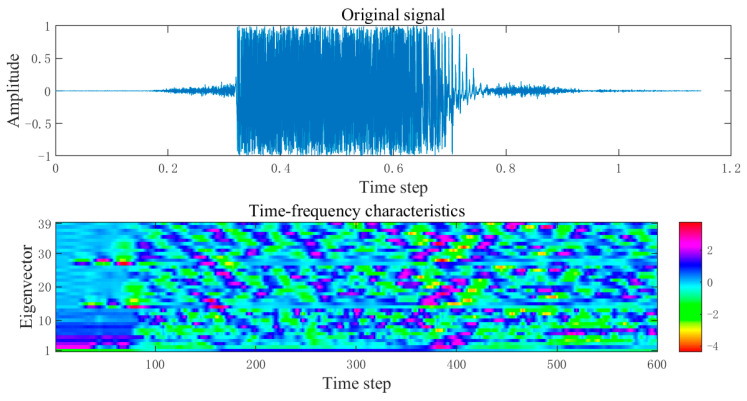
The time–frequency characteristic of the spoken digit “five”.

Taking the spoken digit “five” as an example, its preprocessed time–frequency features are shown in Figure 6.

### 3.3. Spike Encoding Based on Sigma–Delta Modulation

An improved Sigma–Delta modulator is employed to convert continuous feature values into binary spike sequences. This modulator utilizes an integrate–quantize–feedback mechanism to generate sparse spike trains while maintaining a high signal-to-noise ratio. The modulation process can be described by the following difference equation:

(14) $$e\left[n\right]\;=\;x\left[n\right]-y\left[n-1\right]$$

(15) $$s\left[n\right]=s\left[n-1\right]+e\left[n\right]$$

(16) $$q\left[n\right]=\left\{\begin{array}{l} 1,\;if\;s\left[n\right]\geq \theta \\ 0,\;otherwise \end{array}\right.$$

(17) $$y\left[n\right]=q\left[n\right]$$

where $x\left[n\right]$ is the input feature sequence, $y\left[n\right]$ is the output spike sequence, $e\left[n\right]$ is the error signal, $s\left[n\right]$ is the integrator state, and θ is the threshold parameter. The transfer function of this modulator is

(18) $$H\left(z\right)=\frac{Y\left(z\right)}{X\left(z\right)}=\frac{z^{-1}}{1-\left(1-\alpha \right)z^{-1}}$$

where α is the feedback coefficient, which controls the stability of the modulator.

**Figure 7:**
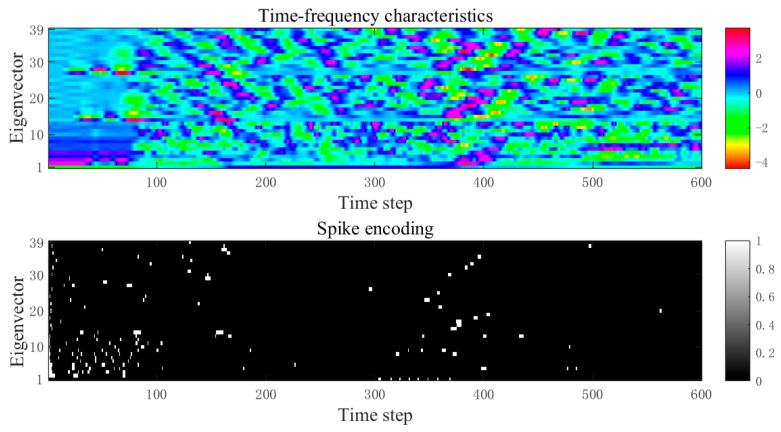
The time–frequency characteristic spike encoding of the spoken digit “five”.

To visually demonstrate the encoding results of the enhanced MFCC-driven sparse spike encoding method on speech signals, taking the spoken digit “five” as an example, its spike encoding result is shown in Figure 7.

**Figure 8:**
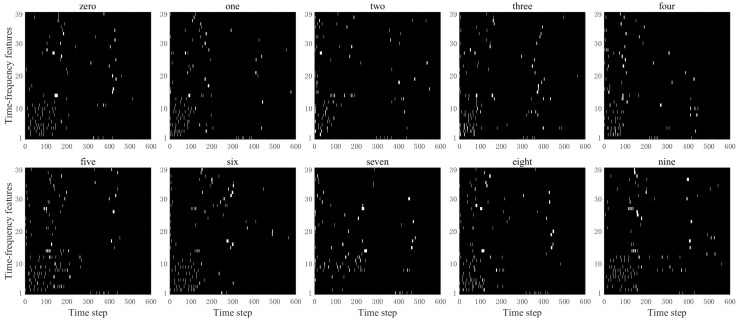
The time–frequency characteristic spiking encoding of different speech signals.

From Figure 7, it can be clearly observed that after spike encoding, the time–frequency features are converted into sparse binary spikes. Spikes are primarily distributed in time segments with relatively significant feature variations, while the number of spikes is notably reduced in regions with gentle changes. This indicates that the proposed enhanced MFCC-driven sparse spike encoding method can preserve the key time–frequency dynamic information of speech while effectively suppressing redundant responses, thereby improving the sparsity and discriminability of the input representation. It provides an input form that aligns with biological neural encoding mechanisms for the efficient processing of subsequent brain-inspired models. The results of converting speech signals “zero” to “nine” into spike sequences are shown in Figure 8.

Figure 8 shows the spike sequences obtained by Sigma–Delta modulation encoding of 39-dimensional time–frequency features of different spoken digits. Each white point represents a spike firing event, visually reflecting the spike response patterns of spoken digits at different time steps and across different time–frequency feature channels. This encoding method converts continuous analog speech features into sparse spike-timing representations, making the input form more compatible with the information processing mechanisms of SNNs, thereby providing a bio-inspired input with a certain degree of biological plausibility for the subsequent temporal computation of SNNs. It can be observed that different spoken digits show relatively distinct differences in terms of spike-timing distribution, response across time–frequency feature channels, and firing density. This indicates that the proposed method effectively preserves the time–frequency structure and dynamic variation information of the original spoken digits while achieving an effective mapping from continuous features to spike representations, providing input representations for spoken digits recognition based on SNNs.

### 3.4. Comparative Evaluation of the Two Preprocessing Methods

To verify the effectiveness of the proposed preprocessing method, we compared the proposed enhanced MFCC-driven sparse spike encoding pipeline with the conventional Lyon ear model + BSA pipeline at the encoding level. Specifically, the comparison was conducted from three aspects: the average number of spikes generated per utterance, the sparsity ratio of the encoded spike representation, and the encoding time required for each utterance. We first compare the average number of spikes generated per utterance for the two preprocessing methods, as shown in Table 3.

Table 3 summarizes the average spikes per utterance produced by the two preprocessing methods for the ten spoken digits. It can be seen that the proposed method generates dramatically fewer spikes than the conventional Lyon + BSA pipeline for all digit categories. For example, for the digit “zero”, the average number of spikes decreases from 5824.64 ± 648.10 to 309.75 ± 30.77, while for “one” it decreases from 5152.31 ± 721.35 to 303.96 ± 28.71. Similar trends are observed for all remaining digits. Overall, the spike count produced by the proposed method is reduced by an order of magnitude compared with the conventional method. This result indicates that the proposed preprocessing strategy can represent spoken-digit signals using a much more compact spike-based code, thereby substantially reducing redundant neural events at the input stage.

We compare the sparsity ratio of the spike representations produced by the two preprocessing methods, as shown in Table 4.

The advantage of the proposed method is further confirmed by the sparsity ratio comparison reported in Table 4. For all ten spoken digits, the proposed method consistently achieves a markedly higher sparsity ratio than the Lyon + BSA pipeline. The sparsity ratios obtained by the conventional method range approximately from 0.874 to 0.932, whereas those of the proposed method are stably concentrated around 0.985–0.987. For instance, for the digit “zero”, the sparsity ratio increases from 0.874 ± 0.0140 to 0.986 ± 0.0013, and for “six”, it increases from 0.932 ± 0.0196 to 0.987 ± 0.0013. These results demonstrate that the proposed method not only reduces the absolute number of spikes but also produces a substantially sparser representation overall. From the perspective of neuromorphic computing, such a representation is highly desirable because sparse spike trains can reduce computational burden and improve energy efficiency in spike-driven systems.

We further compare the encoding time required by the two preprocessing methods, as shown in Table 5.

Table 5 shows that the proposed preprocessing method is consistently faster than the conventional Lyon + BSA pipeline across all digit categories. For example, for the digit “zero”, the encoding time decreases from 31.3 ± 8 ms to 5.4 ± 1 ms, while for “one”, it decreases from 26.5 ± 4 ms to 3.4 ± 0.6 ms. Similar reductions are observed for the remaining digits, with the proposed method typically requiring only about 3.4–5.4 ms per utterance, compared with approximately 25.5–31.3 ms for the conventional method. This substantial reduction in encoding time indicates that the proposed preprocessing strategy provides a clear computational advantage and is therefore more suitable for efficient spike-based speech-processing systems.

## 4. Experimental Section

To evaluate the effectiveness of the proposed preprocessing method, the revised fMRISNN-based framework is applied to a speech recognition task. This section presents two groups of experiments. First, under the same fMRISNN-based speech recognition framework, the proposed enhanced MFCC-driven sparse spike encoding method is compared with the conventional Lyon ear model + BSA pipeline in terms of recognition accuracy. Second, using the proposed preprocessing method, the fMRISNN-based framework is further compared with SNNs using different topologies and with CNN-based models in terms of recognition accuracy, model parameter size, and theoretical energy consumption.

### 4.1. Speech Dataset

The speech recognition experiments were conducted on a subset of the TI46 corpus [37], provided by the Linguistic Data Consortium. This subset contains 4000 utterances of ten isolated spoken digits (“one”–“nine”), produced by 16 speakers (8 male and 8 female), with each digit repeated 25 times per speaker. Of these utterances, 2400 were used for training and the remaining 1600 for testing.

### 4.2. Speech Recognition Framework

**Figure 9:**
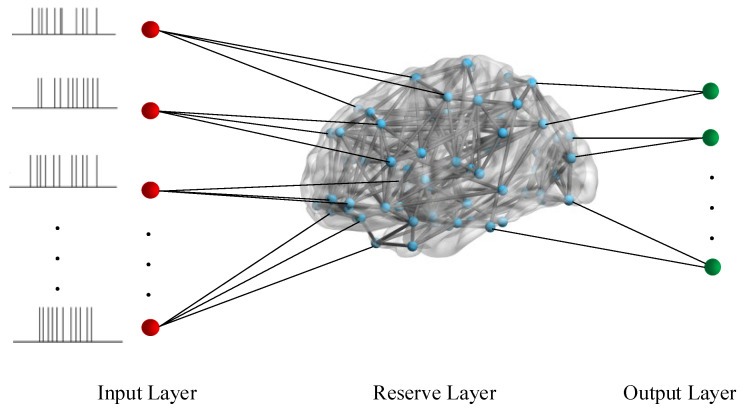
Speech recognition framework based on LSM. (The red circles represent the input speech spike sequences, the blue circles represent the network nodes, namely the neuron models, and the green circles represent the output spike sequences).

This study describes an fMRISNN-based speech recognition framework inspired by the LSM [12]. The framework consists of three components, an input layer, a reservoir layer, and an output layer, as shown in Figure 9.

As shown in Figure 9, the speech recognition framework based on the LSM employs the fMRISNN as the reserve layer. After the preprocessed spikes of spoken digits are fed into the network, the reserve layer is responsible for dynamically mapping and producing high-dimensional representations of the temporal information received by the input layer through the synaptic plasticity model, generating discriminative neural firing activities. On this basis, the connection weights between the reserve layer and the output layer are trained using the Remote Supervised Method (ReSuMe) to adjust the firing activity of the output layer neuron model [38]. Finally, speech category discrimination is completed based on the response patterns of the output layer neuron model.

#### 4.2.1. Neural Firing Activities Under Speech Signals

**Figure 10:**
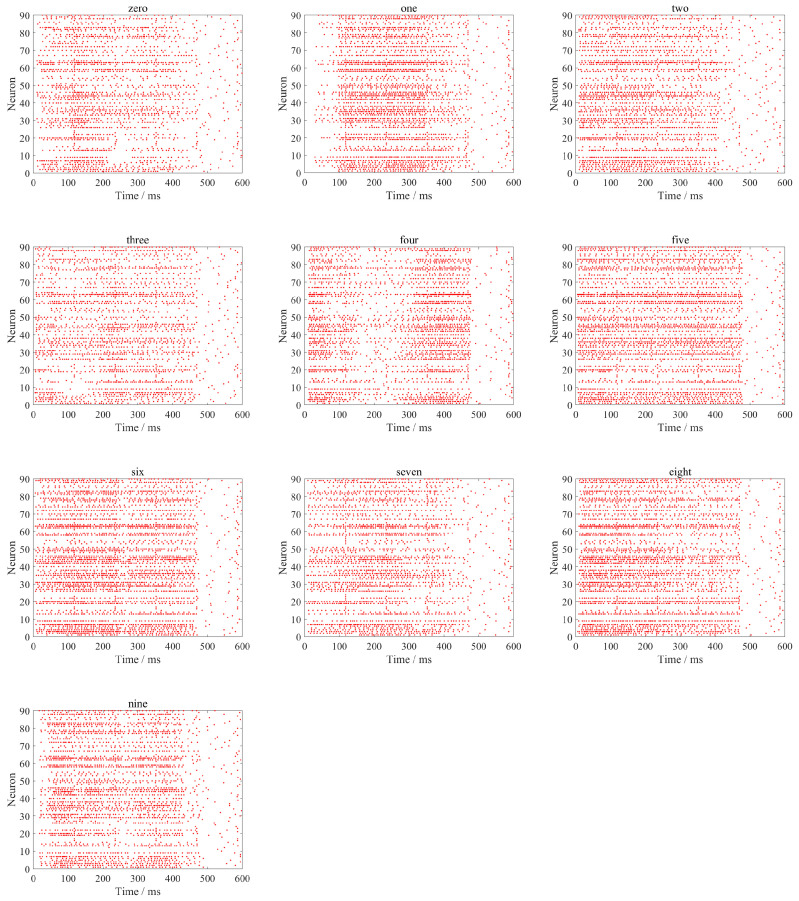
Neural firing activities of fMRISNN under spoken digits.

After the preprocessing stage is completed, the original signals are converted into spikes of spoken digits. These spikes consist of 39 sub-bands, which are randomly fed into the corresponding 39 neuron models in the fMRISNN. For each neuron model receiving the speech signal, the spikes are input into the $I\left(t\right)$ term of the LIF neuron model Equation (2). Figure 10 presents the neural firing activities of fMRISNN under spoken digits over a 600-ms duration, where each red dot indicates a spike firing event of a neuron at the corresponding time.

As shown in Figure 10, the neural firing activities of fMRISNN under different spoken digits exhibit variations. To examine whether the inter-group differences in neural firing activities induced by spoken digits “zero” to “nine” are significant, this study employed a one-way analysis of variance (ANOVA) for analysis. The analysis uses an F-test to evaluate the statistical differences in neural responses under different categories of speech stimuli. The F-value is the ratio of the between-group mean square to the within-group mean square. A larger F-value indicates more significant differences in neuronal firing activities corresponding to different speech categories. The p-value represents the significance level of the F-test, used to determine whether inter-group differences are significant. Typically, p < 0.05 is considered a significant difference, and p < 0.01 a highly significant difference. In this analysis, the number of neural firings evoked by each digit’s speech signals at each time point is defined as a group.

The calculation results show that F = 5.83 and p = 1.71 × 10^−7^ < 0.01, indicating that the neural firing activities of the fMRISNN under different speech signals exhibit a highly significant difference. Based on this finding, this study extracts and utilizes such neural firing activities as features for the speech recognition task.

#### 4.2.2. The Training Process Based on the ReSuMe Algorithm

The connection weights between the reserve and the output layer are trained using the ReSuMe [38]. This method is based on a Hebbian STDP rule, simulating the learning mechanisms of biological neural networks. It has demonstrated excellent performance in temporal signal processing and has been successfully validated in numerous recognition tasks [39]. Therefore, this study employs the ReSuMe method to train the synaptic weights $g\left(t\right)$ between the reserve and the output layer. The update rule is expressed as

(19) $$\frac{dg\left(t\right)}{dt}=\left[S\left(t_{d}\right)-S\left(t_{a}\right)\right]\left[e_{H}+\int_{0}^{\infty }w\left(\Delta t\right)S\left(t_{in}-\Delta t\right)dt\right]$$

The parameter $e_{H}$ was set to 0.25 in this study. The definitions of the terms in this weight update mechanism are as follows: $S(t_{in})$ denotes the input spike sequence; $S(t_{a})$ represents the actual output firing sequence after each training cycle; $S(t_{d})$ denotes the desired output firing sequence, which is defined based on the differences in fMRISNN neural firing activities evoked by different speech data; $e_{H}$ is the weight adjustment coefficient for the Anti-Hebbian term; and w(Δt) is the temporal window modulation function in the STDP rule.

**Figure 11:**
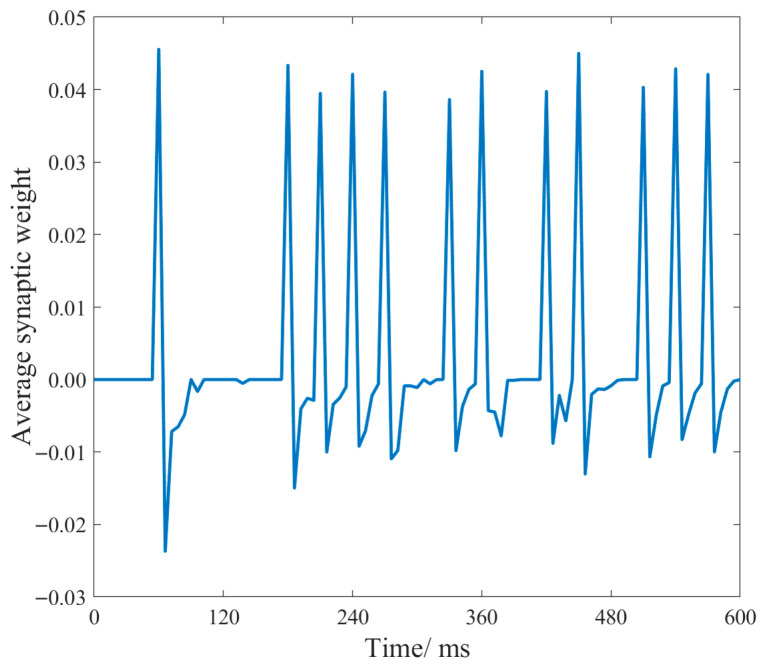
Average synaptic weight changes between the reserve layer and the output layer.

During the training process, every speech signal in the training set is associated with a specific digit. Once the speech recognition framework based on fMRISNN is input with this digit, it is regarded as completing the synaptic weight update for one training cycle. After each digit accumulates through 240 training cycles, the synaptic weights between the reserve pool and the output layer for the corresponding training are obtained. Figure 11 presents the post-training average synaptic weights for the digit “five”.

Figure 11 shows the temporal variation in the average synaptic weights between neurons in the reserve layer (fMRISNN) and the output layer after training is completed. Around the target spike firing times, alternating positive and negative pulsations characterize the average weights between the reserve and output layer neurons. When the weights reach positive peaks, the spiking activity of the output layer neurons is significantly enhanced. This regulatory mechanism drives the actual firing sequence of the output layer neurons to progressively approximate the target spike sequence, thereby achieving improved training outcomes.

### 4.3. The Recognition Performance of fMRISNN

To examine whether the advantages of the proposed preprocessing method in terms of spike number, sparsity, and encoding time are achieved without sacrificing recognition performance, we further conducted a controlled comparison by using the two types of encoded speech inputs within the same fMRISNN-based speech recognition framework described in Section 4.2. The recognition accuracies obtained under the two preprocessing pipelines are summarized in Table 6.

Table 6 shows that the proposed method maintains a recognition performance highly comparable to that of the conventional Lyon + BSA pipeline across all digit categories. For example, the recognition accuracy for “zero” is 97.38% ± 0.57% under the proposed method and 97.50% ± 0.62% under the conventional method, while for “five”, the two methods achieve nearly identical performance, i.e., 95.64% ± 0.57% and 95.62% ± 2.50%, respectively. Similar behavior can be observed for the other digits. Although slight fluctuations exist for individual categories, the overall recognition accuracy remains essentially stable. This indicates that the proposed preprocessing method can substantially reduce spike activity and improve sparsity without sacrificing task-relevant discriminative information.

Taken together, the results in Table 3, Table 4, Table 5 and Table 6 demonstrate that the proposed enhanced MFCC-driven sparse spike encoding method offers clear advantages over the conventional Lyon ear model + BSA pipeline. Specifically, within the same fMRISNN-based speech recognition framework, the proposed method reduces spike number, increases spike sparsity, and shortens encoding time, while maintaining nearly the same spoken-digit recognition accuracy. These results indicate that improving speech input representation can significantly enhance the computational efficiency of SNN-based speech recognition systems without sacrificing recognition performance.

### 4.4. Comparison of Recognition Performance

To investigate the impact of network topology on speech recognition performance, we first compare the proposed fMRISNN with several SNN models featuring different topologies, as presented in Section 4.4.1. On this basis, to further evaluate the overall performance of the proposed framework, we compare it with speech recognition methods reported in the literature and with conventional convolutional neural network (CNN)-based models.

#### 4.4.1. Comparison with SNNs Featuring Different Topologies

The RegSNN, RanSNN, SWSNN, and SFSNN share an identical network scale, neuron model, and synaptic plasticity model with the fMRISNN, with the only difference lying in their topological structures. RegSNN has a regular topology, RanSNN has a random topology, SWSNN has a small-world topology, and the SFSNN has a scale-free topology. The following describes the generation methods for the topological structures of these SNNs.

(1) RegSNN, RanSNN, and SWSNN

We constructed these SNNs based on the Watts–Strogatz algorithm [5]. The implementation steps of this algorithm are show in Algorithm 1.
**Algorithm 1.** Watts–Strogatz small-world network generation.**Input:** Number of nodes *N*, number of nearest neighbors *K*, rewiring probability *p***Output:** A graph *G* with small-world propertiesSteps:1. Construct a regular ring lattice
Create a ring graph with *N* nodes.Connect each node to its *K*/2 nearest neighbors on both sides, resulting in each node having degree *K*.2. Rewire edges with probability *p*For each edge (*u*, *v*) in the original regular graph (considering each edge once), with probability *p*, replace it with a new edge (*u*, *w*), where *w* is randomly selected from all nodes, and self-loops and duplicate edges are avoided.3. Return the generated graph *G.*

**Figure 12:**
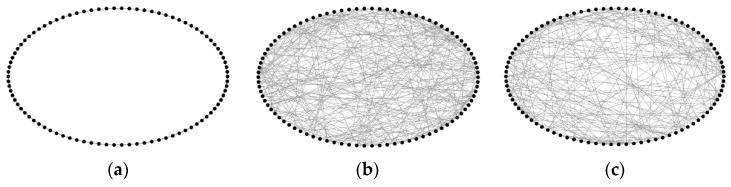
Topological visualization of three SNNs: (**a**) RegSNN, (**b**) RanSNN, and (**c**) SWSNN.

If the rewiring probability *p* = 0, a regular topology is generated; if *p* = 1, a random topology is produced. Their topological structures are illustrated in Figure 12a,b. By setting the rewiring probability within the interval (0, 1), small-world topologies with different *δ* values can be obtained. In this study, the *δ* value of fMRISNN is 1.76. For comparison, we adjusted parameters to construct an SWSNN with the same *δ* value, whose topological structure is shown in Figure 12c.

(2) SFSNN

To obtain a scale-free topology with power-law degree distribution, the SFSNN was constructed based on the Barabási–Albert algorithm [6]. The implementation steps of this algorithm are shown in Algorithm 2.
**Algorithm 2.** Algorithm: Barabási–Albert scale-free network generation.**Input:** Initial number of nodes $m_{0}$, number of edges to attach per new node $m_{e}$ ($m_{e}$ $\leq m_{0}$), total number of nodes *N***Output:** A scale-free graph *G* with power-law degree distributionSteps:1. Initialization
Start with a fully connected graph consisting of $m_{0}$ nodes2. Growth and preferential attachmentFor each new node i from $m_{0}$ + 1 to *N*: Add node i to the network; connect node i to $m_{e}$ existing nodes, selected with probability proportional to their current degree; ensure no multiple edges or self-loops.3. Return the generated graph *G.*

**Figure 13:**
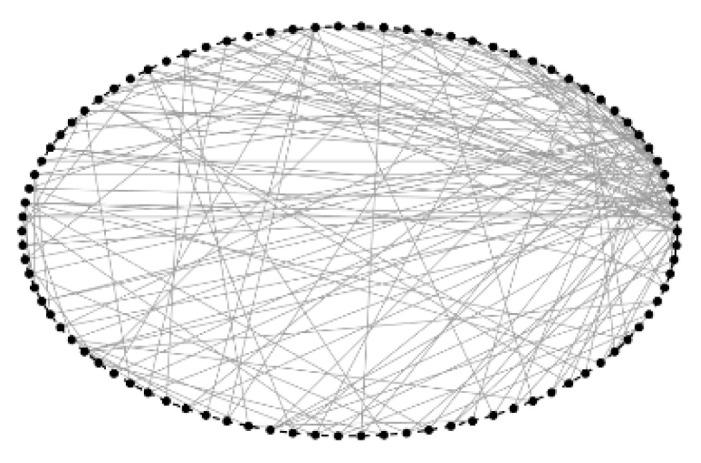
Topological visualization of SFSNN.

Scale-free topologies with different degree exponents *γ* can be obtained by tuning the network parameters $m_{0}$ and $m_{e}$. In this study, the *γ* value of fMRISNN is 2.08; we adjusted the corresponding parameters to generate an SFSNN with the same *γ* value for comparison. Its topological structure is shown in Figure 13.

To assess and compare the recognition accuracy of the SNNs described above, we carried out speech recognition experiments as detailed in Section 4.2. Table 7 shows the accuracy rates for these SNNs, as well as the mean and standard deviation of the accuracy rates for the fMRISNN across the 10 subjects.

Table 7 shows that fMRISNN achieves the highest speech recognition accuracy among all compared models. In general, the complex-network-based SNNs (SWSNN, SFSNN, and fMRISNN) outperform the non-complex models (RegSNN and RanSNN), indicating that network topology plays an important role in recognition performance. Among them, fMRISNN yields the best result, which suggests that an SNN architecture derived from biologically plausible functional brain networks is more effective for speech recognition than manually designed topologies.

**Figure 14:**
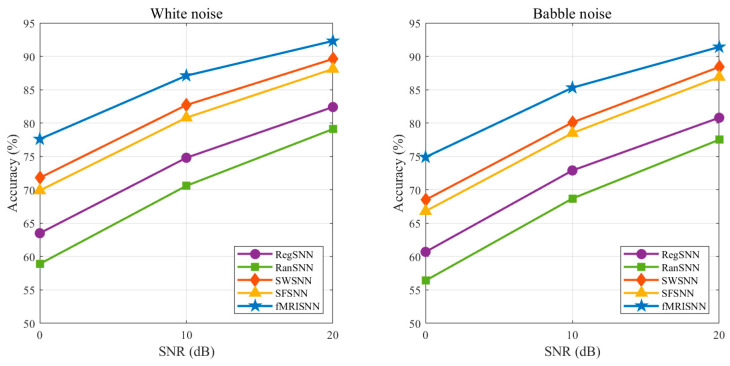
Recognition accuracy of SNNs under additive noise conditions.

To evaluate the robustness of different SNNs under noisy conditions, additive noise was introduced into the test speech signals. Two types of noise were considered: white noise and babble noise. For each noise type, noisy speech samples were generated at signal-to-noise ratios (SNRs) of 20 dB, 10 dB, and 0 dB. All models were trained under the same setting as in the clean-condition experiment and were then evaluated on the corresponding noisy test sets. Recognition accuracy was used as the evaluation metric. The results are shown in Figure 14.

As shown in Figure 14, the recognition accuracy of all models decreased as the noise level increased. However, fMRISNN consistently achieved the highest accuracy across all noise conditions. In particular, under both white noise and babble noise, the performance degradation of fMRISNN was smaller than that of the baseline models, indicating stronger robustness to acoustic interference. This result further supports that the biologically plausible topology derived from FBNs benefits not only recognition accuracy in clean conditions but also noise-resistant speech processing.

#### 4.4.2. Comparison with Related Speech Recognition Methods

To further assess the performance of the proposed fMRISNN-based speech recognition framework, we compared it with related SNN-based speech recognition methods reported in the literature. Because the existing studies differ in learning algorithms, dataset configurations, and experimental protocols, a rigorous one-to-one comparison is not feasible. Therefore, recognition accuracy was adopted as the main metric for comparative evaluation.

The research team constructed various topological structures of SNNs and evaluated their speech recognition performance on the 5, 8, and 16 speaker subsets of the TI46 speech dataset. To maintain consistency in comparison, this paper constructs a speech recognition experiment based on fMRISNN using the same three subsets, thereby achieving a direct comparison with existing SNN methods in terms of recognition accuracy. The speech recognition accuracies of each method are shown in Table 8.

Table 8 shows the superior recognition accuracy of our method over the reported methods. Although on the 5-speaker dataset, the recognition accuracy of our model is basically the same as that of the method in reference [9], it is significantly better than the latter on the 16-speaker dataset. This demonstrates that our method exhibits stronger robustness on more complex speech recognition tasks.

#### 4.4.3. Comparison with the Recognition Performance of CNN

**Figure 15:**
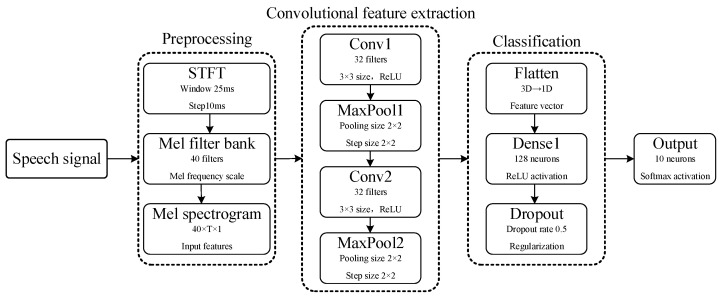
Speech recognition framework based on CNN.

To provide a more comprehensive evaluation of the proposed fMRISNN model, we further compared it with a conventional CNN-based speech recognition model on the TI46 spoken-digit recognition task. The CNN-based speech recognition framework used in this section is shown in Figure 15.

Firstly, the input speech signal is preprocessed: the short-time Fourier transform (STFT) is used to extract time–frequency features, with a window length of 25 ms and a step size of 10 ms; then the signal passes through a Mel filter bank consisting of 40 filters and is converted to the Mel-frequency scale, finally producing a 40 × T × 1 Mel spectrogram as the input feature, where T denotes the total number of time frames in the speech sample. Subsequently, the feature map is successively passed through two convolutional modules: the first module contains 32 convolution kernels of size 3 × 3, followed by a ReLU activation function and a 2 × 2 max pooling layer; the second module contains 64 convolution kernels of size 3 × 3, also using the ReLU activation function and 2 × 2 max pooling. Finally, the features are flattened into a one-dimensional vector through the Flatten layer, connected to a fully connected layer with 128 neurons (ReLU activation) and regularized using Dropout (dropout rate 0.5), ultimately outputting the classification result.

We used the same training set and test set to conduct a comparative experiment between fMRISNN and the aforementioned CNN on the TI46 speech digital recognition task. We conducted all simulations using MATLAB R2023a on a system equipped with a 13th Gen Intel(R) Core (TM) i7-13620H processor (2.40 GHz) and 16.0 GB of RAM, without the use of a GPU. The experimental results are shown in Table 9.

Table 9 shows that the fMRISNN achieves a recognition accuracy similar to that of CNN on the test set. Although CNN has strong expression capabilities in feature extraction and pattern recognition, fMRISNN, by introducing FBN connection constraints, constructs a topologic structure that is closer to bio-plausibility and can also achieve effective recognition of speech digits. In addition, fMRISNN outperforms traditional CNN in terms of model parameter size, energy consumption, and bio-plausibility. CNN usually relies on large-scale parameters and high computational complexity to achieve high performance, while fMRISNN, through sparse connections and pulse dynamics, maintains a lower computational resource requirement while also having greater practical application potential in neuromorphic computing.

## 5. Discussion

To better understand why the fMRISNN achieves superior performance in speech recognition, this section analyzes the model from two perspectives: neural firing activity and neural information transmission.

### 5.1. Neural Firing Activity

Given that speech recognition accuracy strongly depends on neural firing activities (Section 4.2.1), we performed a one-way ANOVA to examine firing differences across the SNNs described in Section 4.4.1 and the proposed fMRISNN under speech signals. Table 10 reports the corresponding p-values and F-values, whose definitions are provided in Section 4.2.1.

Table 10 shows that the fMRISNN exhibits the most significant differences in neural firing activities under speech signals, consistent with its superior recognition accuracy. All SNNs show highly significant firing pattern differences (p < 0.01), confirming their pattern discrimination capability. Furthermore, F-value analysis demonstrates that complex SNNs (SWSNN, SFSNN, and fMRISNN) outperform non-complex SNNs (RegSNN and RanSNN), and biologically plausible SNNs (fMRISNN) outperform non-biologically plausible SNNs—aligning with the accuracy trends observed in Table 7.

### 5.2. Neural Information Transmission

Neural firing activity propagates via synapses along the network topology. To explain the observed firing differences among SNNs, we analyze their neural information transmission mechanisms in terms of synaptic density and dynamic topological properties.

#### 5.2.1. Synaptic Density

**Figure 16:**
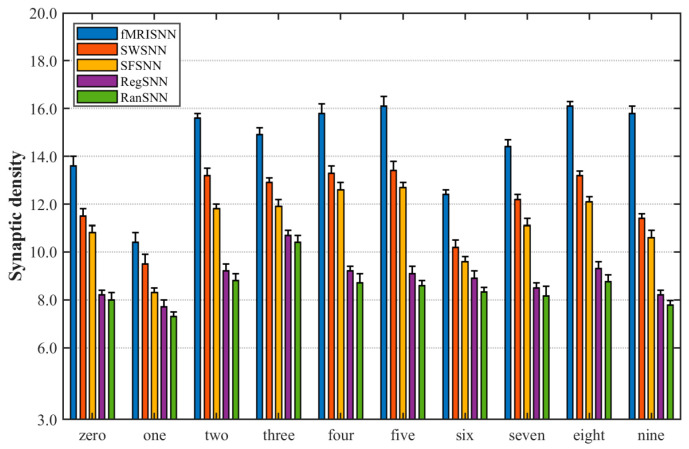
Synaptic density of SNNs with different topologies under speech signals.

Synaptic density measures active synapses per neuron (non-zero weights enabling firing transmission) [44]. To analyze the reasons for the differences in neural firing among these SNNs described in Section 4.4.1 and our fMRISNN, we calculated their synaptic density under speech stimulation. The results are shown in Figure 16.

Figure 16 shows that the fMRISNN exhibits the highest synaptic density, followed by other complex and biologically plausible SNNs, which outperform their non-complex and non-biologically plausible SNNs. This indicates that SNNs with higher synaptic density possess more information transmission pathways, thereby leading to more active neural firing activity.

#### 5.2.2. Dynamic Topological Properties

Neural firing transmission is shaped by topological structure. We therefore investigated how variations in the clustering coefficient (CC) and shortest path length (SPL) across SNNs account for their distinct firing patterns.

(1) CC

The CC is used to describe the degree of clustering among nodes in a network, and its value reflects the efficiency of local information transmission within the network. Typically, a higher CC indicates stronger local information transmission capability in the network. Since fMRISNN is a dynamically weighted network, this study employs the weighted average CC (denoted as $C_{w}$) [45], defined as follows:

(20) $$C_{w}\;=\;\frac{1}{N}\sum_{i=1}^{N}\left(\frac{1}{s_{i}\left(D_{i}-1\right)}\sum_{j,k}\frac{\left(g_{ij}+g_{ik}\right)}{2}a_{ij}a_{jk}a_{ki}\right)$$

where N represents the number of neuron models; $s_{i}$ and $D_{i}$ denote the node strength and node degree of the neuron model i, respectively; $g_{ij}$ and $g_{ik}$ are synaptic weights; and $a_{ij}$, $a_{jk}$ and $a_{ki}$ are elements of the adjacency matrix.

(2) SPL

The SPL characterizes the minimum number of edges required to traverse between any two nodes in a network and can be used to evaluate the global information transmission efficiency of the network. Generally, a lower value of the SPL indicates higher global information transmission efficiency. This study adopts the weighted average SPL (denoted as $L_{w}$) [45], defined as follows:

(21) $$L_{w}\;=\;\frac{1}{N\left(N-1\right)}\begin{array}{c} min\\ \delta \left(i,j\right)\in \Gamma \left(i,j\right) \end{array}\left[\sum_{m,n\in \delta \left(i,j\right)}\frac{1}{g_{mn}}\right]$$

where $g_{mn}$ represents the synaptic weight; $\delta \left(i,j\right)$ denotes a path from neuron model i to neuron model j; and $\Gamma \left(i,j\right)$ represents the set of all possible paths between neuron model i and neuron model j.

**Figure 17:**
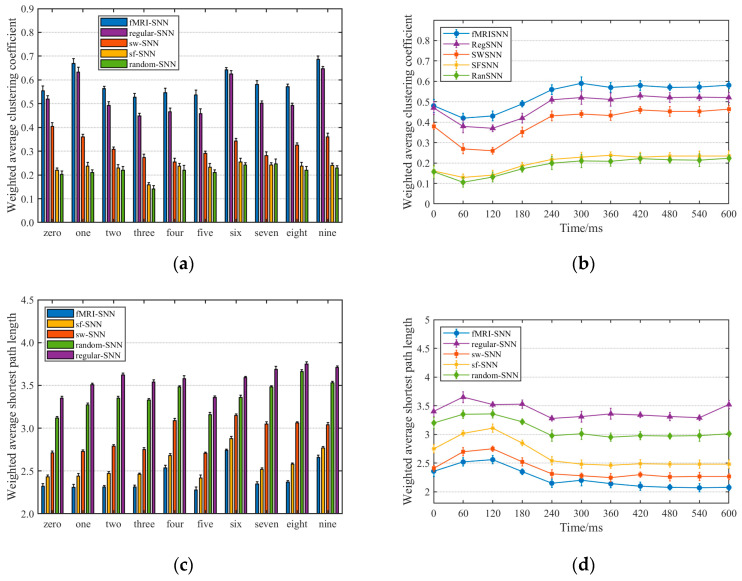
Dynamic topological features of SNNs under speech signals: (**a**) Weighted average CC; (**b**) Dynamic weighted average CC of the speech signal “five”; (**c**) Weighted average SPL; (**d**) Dynamic weighted average SPL of the speech signal “five”.

Based on $C_{w}$ and $L_{w}$, we further compared the neural information transmission of SNNs with different topologies under various speech signals. The results of $C_{w}$ and $L_{w}$ for the five SNNs are presented in Figure 17a and Figure 17c, respectively. To analyze the evolution of network topology during training, we examined the changes in the topological features $C_{w}$ and $L_{w}$ at different training stages. Taking the speech signal for “five” as an example, the evolution of $C_{w}$ and $L_{w}$ over the training process is illustrated in Figure 17b and Figure 17d, respectively.

Figure 17 shows that fMRISNN achieves the highest weighted CC and the lowest weighted SPL, indicating optimal global information transmission efficiency. This superiority is supported by two theoretical perspectives: Non-complex SNNs (RegSNN and RanSNN) fail to balance local and global transmission efficiency—RegSNN has high clustering but long paths, while RanSNN has short paths but low clustering. Complex SNNs (SWSNN, SFSNN, and fMRISNN) achieve better balance, outperforming non-complex networks; biologically plausible SNNs (fMRISNN) exhibit higher CCs and SPLs than those lacking bio-plausibility, confirming their superior transmission efficiency in both local and global information processing.

In summary, the comprehensive analysis of synaptic density, average weighted CC, and average weighted SPL indicates that the information transmission capability of SNNs with complex network structures is superior to that of SNNs with non-complex network structures; the information transmission capability of biologically plausible SNNs is superior to that of SNNs lacking bio-plausibility. Specifically, fMRISNN possesses the optimal information transmission capability. Since neural firing activity is transmitted through synapses within the topological connectivity structure, the performance of neural firing activity largely depends on the information transmission capability of the SNN. These findings provide theoretical support for the comparative results of neural firing activity among the various SNNs examined in this study.

## 6. Conclusions

This study revisits the fMRISNN framework for speech recognition from the perspective of speech preprocessing and spike encoding. Specifically, an enhanced MFCC-driven sparse spike encoding method is proposed and compared with the conventional Lyon ear model + BSA pipeline to examine whether improved input representation can enhance performance within the same fMRISNN-based speech recognition framework. Experimental results show that the proposed preprocessing method reduces spike number, increases spike sparsity, and shortens encoding time, while maintaining nearly the same recognition accuracy. In addition, the fMRISNN remains competitive when compared with SNN models using different topologies and with a conventional CNN-based model. These findings indicate that improved speech preprocessing is an effective way to enhance the practical efficiency of SNN-based speech recognition systems without sacrificing recognition capability. They also suggest that the performance of the fMRISNN depends not only on biologically constrained topology but also on the quality and efficiency of the input spike representation. Nevertheless, the present study is limited to the spoken-digit recognition task and a relatively restricted experimental setting. Broader validation on additional datasets and speech-related tasks is therefore needed, which will be an important direction for future work.

## Figures and Tables

**Table 1:** The parameters of the LIF neuron.

Parameter	Value	Instruction
$\tau_{m}$	20 ms	Membrane time constant
$V_{\mathrm{rest}}$	−65 mv	Resting potential
$V_{\mathrm{th}}$	−50 mv	Firing threshold
$V_{\mathrm{reset}}$	−70 mv	Reset potential
$R_{m}$	1 MΩ	Membrane resistance
$\tau_{\mathrm{ref}}$	2 ms	Refractory period

**Table 2:** The parameters of the synaptic plasticity model.

Parameter	Instruction	Value
$E_{syn}$	Synaptic reversal potential	Excitatory: 0 mV
		Inhibitory: −70 mV
α	Forward neurotransmitter release rate constant	Excitatory: 2
		Inhibitory: 0.9
β	Reverse neurotransmitter release rate constant	Excitatory: 1
		Inhibitory: 0.1
τ	Synaptic delay	0.1–40 ms, following a Poisson distribution.
$\tau_{ex}$	Excitatory synaptic decay constant	3 ms
$\tau_{in}$	Inhibitory synaptic decay constant	5 ms
$g_{max}$	Maximum synaptic weight	0.015
$A_{+}$	Excitatory synaptic long-term potentiation (LTP) magnitude	0.1
$A_{-}$	Excitatory synaptic long-term depression (LTD) magnitude	0.105
$B_{+}$	Inhibitory synaptic LTP magnitude	0.02
$B_{-}$	Inhibitory synaptic LTD magnitude	0.03
$\tau_{+}$	Effective presynaptic/postsynaptic firing time window for triggering LTP	20 ms
$\tau_{-}$	Effective presynaptic/postsynaptic firing time window for triggering LTD	20 ms

**Table 3:** Comparison of the average spikes per utterance of the two preprocessing methods.

Spoken Digits	Avg. Spikes/Utterance (Lyon + BSA)	Avg. Spikes/Utterance (Proposed)
zero	5824.64 ± 648.10	309.75 ± 30.77
one	5152.31 ± 721.35	303.96 ± 28.71
two	5073.66 ± 672.89	313.58 ± 32.56
three	4980.04 ± 663.03	309.74 ± 29.99
four	4942.34 ± 644.81	326.79 ± 35.10
five	5147.14 ± 691.65	308.62 ± 33.41
six	3151.51 ± 907.14	300.47 ± 31.55
seven	4422.32 ± 589.90	349.36 ± 37.41
eight	3567.24 ± 597.28	287.66 ± 36.33
nine	5746.81 ± 618.18	302.18 ± 29.06

*Note: Values are reported as mean ± standard deviation. “Lyon + BSA” denotes the conventional preprocessing pipeline based on the Lyon ear model and the BSA. “Proposed” denotes the enhanced MFCC-driven sparse spike encoding method introduced in this study. For each spoken digit, the average number of spikes was calculated over all utterances belonging to that category.*

**Table 4:** Comparison of the sparsity ratio of the two preprocessing methods.

Spoken Digits	Sparsity Ratio (Lyon + BSA)	Sparsity Ratio (Proposed)
zero	0.874 ± 0.0140	0.986 ± 0.0013
one	0.888 ± 0.0156	0.987 ± 0.0012
two	0.890 ± 0.0146	0.986 ± 0.0013
three	0.892 ± 0.0144	0.986 ± 0.0012
four	0.893 ± 0.0140	0.986 ± 0.0015
five	0.889 ± 0.0150	0.986 ± 0.0014
six	0.932 ± 0.0196	0.987 ± 0.0013
seven	0.904 ± 0.0128	0.985 ± 0.0015
eight	0.924 ± 0.0129	0.987 ± 0.0015
nine	0.876 ± 0.0134	0.987 ± 0.0012

*Note: Values are reported as mean ± standard deviation. The sparsity ratio denotes the proportion of zero-activity units in the encoded spike representation, where a higher value indicates a sparser encoding result. For each spoken digit, the reported value was calculated over all utterances belonging to that category.*

**Table 5:** Comparison of the encoding time of the two preprocessing methods.

Spoken Digits	Encoding Time/Utterance (Lyon + BSA)	Encoding Time/Utterance (Proposed)
zero	31.3 ± 8 ms	5.4 ± 1 ms
one	26.5 ± 4 ms	3.4 ± 0.6 ms
two	25.5 ± 3 ms	3.4 ± 0.5 ms
three	27.2 ± 5 ms	3.6 ± 0.7 ms
four	26.8 ± 4 ms	3.8 ± 0.6 ms
five	28.3 ± 6 ms	4.1 ± 0.9 ms
six	31.3 ± 8 ms	4.2 ± 0.8 ms
seven	29.2 ± 7 ms	3.7 ± 0.7 ms
eight	26.9 ± 5 ms	3.6 ± 0.5 ms
nine	29.8 ± 6 ms	3.7 ± 0.7 ms

*Note: Values are reported as mean ± standard deviation. Encoding time was measured for a single utterance under the same experimental environment.*

**Table 6:** Comparison of the two preprocessing methods under the same fMRISNN-based speech recognition framework.

Spoken Digits	Recognition Accuracy (Lyon + BSA) [16]	Recognition Accuracy (Proposed)
zero	97.50% ± 0.62%	97.38% ± 0.57%
one	96.56% ± 1.56%	96.15% ± 0.82%
two	96.25% ± 0.63%	95.58% ± 0.84%
three	89.69% ± 0.31%	88.72% ± 0.64%
four	85.31% ± 0.31%	85.43% ± 0.81%
five	95.62% ± 2.50%	95.64% ± 0.57%
six	94.06% ± 2.18%	94.26% ± 0.18%
seven	97.19% ± 0.31%	97.03% ± 0.53%
eight	96.25% ± 1.25%	96.14% ± 0.72%
nine	96.25% ± 1.25%	96.12% ± 0.47%

*Note: Values are reported as mean ± standard deviation. Recognition accuracy was evaluated under the same speech recognition framework, with the preprocessing pipeline being the only changed component.*

**Table 7:** Recognition accuracy comparison of SNNs.

Networks	Topology	Accuracy
RegSNN	regular	86.21%
RanSNN	random	82.73%
SWSNN	small-world	92.35%
SFSNN	scale-free	90.86%
fMRISNN	FBN	94.25% ± 0.62%

**Table 8:** Comparison with related methods.

Networks	No. of the Speakers	Accuracy
LSM based on recurrent SNN [40]	5	97.50%
LSM based on random SNN [41]	5	98.00%
SWTA-SNN [42]	8	95.25%
SNN with ST-DFA [43]	16	84.88%
LSM based on SWSNN [15]	16	92.38%
Digital LSM based on SNN [12]	5	99.79%
	16	92.30%
fMRISNN	5	99.16 ± 0.41%
	8	97.43 ± 0.27%
	16	94.25 ± 0.62%

**Table 9:** Comparison of recognition performance with the CNN.

Networks	No. of the Speakers	Accuracy	# Parameters	Energy Consumption
CNN	5	99.57%	≈684 k	≈5.0 mJ
	8	98.74%		
	16	96.18%		
fMRISNN	5	99.16 ± 0.41%	≈4 k	≈1.0 mJ
	8	97.43 ± 0.27%		
	16	94.25 ± 0.62%		

*Note: “#” denotes “number of.”.*

**Table 10:** Comparison of p-values and F-values for SNNs with different topologies.

	RegSNN	RanSNN	SWSNN	SFSNN	fMRISNN
p-values	3.51 × 10^−4^	6.41 × 10^−5^	5.72 × 10^−7^	4.49 × 10^−5^	1.37 × 10^−7^
F-values	3.04	3.27	5.53	3.88	5.96

## Data Availability

The original contributions presented in this study are included in the article. Further inquiries can be directed to the corresponding author. Complete code reproducing simulation results written in MATLAB is publicly available at https://github.com/syhtsr/fMRI-SNN.git (accessed on 22 April 2026).

## Abbreviations

fMRI	functional magnetic resonance imaging
BOLD	blood oxygenation level-dependent
SNN	spiking neural network
BSA	Bens Spiker algorithm
FIR	finite impulse response
DTW	dynamic time warping
FBN	functional brain network
LIF	leaky integrate-and-fire
LSM	liquid state machine
MFCC	Mel-frequency cepstral coefficient
ReSuMe	remote supervised method
STDP	spike-timing-dependent plasticity
CNN	convolutional neural network
